# The association of IL-17A rs2275913 single nucleotide polymorphism with anti-tuberculous drug resistance in patients with pulmonary tuberculosis

**DOI:** 10.1186/s43141-023-00542-5

**Published:** 2023-09-04

**Authors:** Asmaa A. Elmadbouly, Abeer Mohammed Abdul-Mohymen, Heba H. Eltrawy, Hanaa A. Abou Elhasan, Azza Ali Althoqapy, Doaa R. Amin

**Affiliations:** 1 Clinical Pathology Department, Faculty of Medicine (Girls), Al-Azhar University, Cairo, Egypt; 2 Chest Diseases Department, Faculty of Medicine (Girls), Al-Azhar University, Cairo, Egypt; 3 Community Medicine Department, Faculty of Medicine (Girls), Al-Azhar University, Cairo, Egypt; 4 Medical Microbiology and Immunology Department, Faculty of Medicine (Girls), Al-Azhar University, Cairo, Egypt; 5 Biochemistry Department, Faculty of Medicine (Girls), Al-Azhar University, Cairo, Egypt

**Keywords:** Tuberculosis, Drug-resistant, Drug-sensitive, Single nucleotide polymorphism, Interleukin-17, IL 17–197 G > A (rs2275913)

## Abstract

**Background:**

Drug-resistant  Tuberculosis (DR-TB) is a global health burden with high morbidity and mortality in developing countries including Egypt. The susceptibility to infection with DR-TB strains may be genetically determined. Several interleukin gene polymorphisms were investigated as risk factors for tuberculosis infection but focusing on their association with DR-TB was limited. Therefore, the objective of this study is to assess the association of IL 17 − 197 G > A (rs2275913) single nucleotide polymorphism (SNP) with susceptibility to DR-TB strains in comparison to drug-sensitive tuberculosis (DS-TB) strains in Egyptian patients with pulmonary TB. This cross-sectional study was conducted on 80 patients with DR-TB strains and 80 with DS-TB strains as a control group. Both age and sex were comparable among the study’s groups. IL-17 − 197 G > A (rs2275913) SNP was genotyped by real-time PCR, and IL-17 serum concentration was measured by enzyme-linked immunosorbent assay (ELISA).

**Results:**

The GA and AA genotype frequencies of IL 17 − 197 G > A (rs2275913) SNP were significantly higher in patients with DR-TB strains than those with DS-TB strains (*p* < 0.001). The frequency of the A allele was significantly (*p* < 0.001) higher in patients with DR-TB group (32.5%) compared to the control group (13.8%). Substantial higher serum levels of IL-17 were detected in the DR-TB group with significant association with AA and AG genotypes.

**Conclusion:**

Polymorphism in IL-17 -197 G > A (rs2275913) resulted in higher serum levels of IL-17 and Egyptian patients with such polymorphism are three times at risk of infection with DR-TB strains than patients with wild type.

## Background

Tuberculosis (TB) is still a global health problem especially in developing countries, where it results in a high morbidity and mortality [1]. The problem is exaggerated by the increasing prevalence of drug-resistant tuberculosis (DR-TB), in which the treatment is characterized by high cost, longer time, lower effectiveness, and more side effects [2]. In Egypt, TB is ranked as the third most prevalent infectious disease after Bilharziasis and hepatitis C [3]. There is a growing concern regarding the rising incidence of multidrug-resistant TB (MDR-TB), characterized by resistance to isoniazid (INH) and rifampicin (RIF), and extensively drug-resistant TB (XDR-TB), which involves resistance to INH, RIF, and at least one fluoroquinolone and one other second-line drug [4, 5].

The World Health Organization (WHO) reported that 3.3% of the newly diagnosed TB cases and 18% of recurrent cases worldwide in 2019 are MDR-TB, which accounts for about 500,000 patients worldwide [6].

Studying the molecular components of immunopathology can help introducing new therapeutics to decrease tissue damage, lower morbidity, and mortality and limit further dissemination. Furthermore, this may offer an adjuvant to anti-tuberculous therapy, reducing the therapy duration and ameliorating drug resistance [7].

Genetic polymorphisms with interleukin activation may interfere with the susceptibility to different TB strains through destabilization of the immune response [8].

IL-17A is a proinflammatory cytokine that plays a crucial role in the immune response to intracellular pathogens [9]*.* The gene for IL-17A is located on chromosome 6p12, and it is produced mainly by Th17 cells, as well as other immune cells [10, 11].

IL-17 -197 G > A (rs2275913) SNP is caused by a replacement of the G by A nucleotide base in the IL-17A gene promoter, resulting in variations in IL-17A production [12].

Identifying individuals who are genetically predisposed to tuberculosis is valuable for personalized treatment. If such individuals can be identified, they may require altered vaccination strategies, heightened vigilance in case of exposure to TB, and prophylactic treatment to reduce their risk [13].

Although several studies have investigated the associations of IL-17 polymorphisms with susceptibility to infection with mycobacterium tuberculosis (MTB) strains [14–18], No research investigating their specific effect on the susceptibility to infection with DR-TB strains has been conducted in Egypt. Therefore, this study aimed to assess the association of IL-17 -197 G > A (rs2275913) SNP with the susceptibility to infection with DR-TB strains compared to the susceptibility to infection with DS-TB strains in Egyptian patients with pulmonary TB.

## Methods

### Study design and participants

This comparative cross-sectional study was conducted at the outpatient clinic of the Chest Diseases Department, Al-Zahraa University Hospital, Cairo, Egypt, from January to June 2021. The study was done in compliance with the Helsinki Declaration 2013 and was approved by the research ethics committee of the Faculty of Medicine for Girls, Al-Azhar University (IRB: 202103775). All patients gave written informed consent before they participated in the study. The study classified patients into two groups: DR-TB and DS-TB. We included both male and female individuals aged 18 years old or above.

DR-TB patients were identified on the basis of the GeneXpert MTB/RIF test results. Those with resistance to rifampicin were included in the drug-resistant group, while those with negative results were classified as drug-sensitive. The patients in the DR-TB group were further categorized into single, multi-drug, or extensively DR-TB based on the results of manual susceptibility testing with various anti-TB drugs. Single-drug resistance was defined as resistance to rifampicin only, multi-drug resistance as resistance to both isoniazid and rifampicin, and extensively drug resistance as resistance to fluoroquinolones, and second-line injectable drugs, such as capreomycin, kanamycin, and amikacin.

#### Inclusion and exclusion criteria

All subjects with recently diagnosed pulmonary TB (based on clinical presentation, radiological findings, and microbiological testing) before the initiation of their treatment and agreed to participate in the study were eligible. Those with autoimmune diseases, cancer, human immunodeficiency virus (HIV), latent TB, bacterial or viral infections, and previous history of anti-tuberculous drug use were excluded.

### Sample size

Since there was a lack of published research on the association of the IL-17-197 G > A (rs2275913) SNP with anti-TB drug resistance, we based our assumption on data from a previous study that reported a 33% frequency of the SNP in all TB patients. We estimated the prevalence of this polymorphism in the DR-TB group to be 40% and in the DS-TB group to be 20% [12]. We used the following formula for the sample size calculation [19]: *n* = (Zα/2 + Zβ)2 * (p1(1-p1) + p2(1-p2))/(p1-p2)2, where Zα/2 is the critical value of the normal distribution at α/2 (e.g., for a 95% confidence level, *α* is 5% and the critical value is 1.96), Zβ is the critical value of the normal distribution at *β* (e.g., for a power of 80%, *β* is 20% and the critical value is 0.84) and p1 and p2 are the expected frequencies of the gene of the two groups, respectively. The formula revealed *n* = 78.4 to be rounded to 80. The study had a sample size of 160 patients, with an allocation ratio of 1:1.

### Data collection

The following data were collected during the study: demographic information of the patients, details of their medical history, results of physical examination, laboratory results including: Ziehl–Neelsen staining and GeneXpert MTB/RIF assay, culture tests, susceptibility tests, and findings from chest X-rays.

### Laboratory investigations

Five milliliters of the peripheral blood were obtained by venipuncture from every participant at the time of TB diagnosis and before the initiation of treatment. Three milliliters were placed in a gel separator vacutainer tube from which the serum was separated by centrifugation and used for routine laboratory tests while the remaining serum was stored at − 20 °C for the enzyme-linked immunosorbent assay (ELISA) measuring of IL-17 level. The remaining 2 ml were put in a vacutainer tube containing EDTA and used for complete blood count and ESR measurement, with the storage of the remaining blood at − 20 °C till the time of SNP genotyping.

Routine laboratory tests were done included complete blood count using the cell counter Cell dyne Ruby, Abbott, Germany, and Biochemical parameters (blood urea, serum creatinine, AST, and ALT) using the chemistry auto-analyzer machine (Cobas Integra 400 plus, Roche diagnostics, Germany).

Serum concentrations IL-17A were measured by sandwich ELISA (Biotech Co., Ltd., Shanghai, China, cat. no. E0142Hu) following the manufacturer’s instructions.

### DNA isolation

The total genomic DNA was extracted using [QIAamp DNA Blood Mini Kit] (Qiagen, Germany, cat. no. 51304) according to the kit protocol followed by measuring the DNA concentration using the [Nanodrop spectrophotometer] (Thermo Scientific-GE). In brief, 20 µl of proteinase K was added to 200 UL of EDTA whole blood and 200 ul of lysis buffer (AL) in a 1.5 ml microcentrifuge tube and vortexed for 15 s followed by incubation at 56 °C for 10 min. After centrifugation, 200 µl absolute ethanol was added to the sample and vortexed again. After another centrifugation, the mixture was placed in the QIAamp Mini spin column and centrifuged at 8000 rpm for 1 min. Then, 500 µl of buffer AW1 was added and recentrifuged at 8000 rpm for 1 min. A second wash was done by adding 500 µl of buffer AW2 followed by centrifugation at 14,000 rpm for 3 min. Finally, the elution of DNA was done in 200 µl of buffer AE after incubation at room temperature (15–25 °C) for 1 min and then centrifugation at 8000 rpm for 1 min.

### Genotyping of IL 17 -197 G > A (rs2275913) SNP

Polymorphism of IL 17 − 197 G > A (rs2275913) was genotyped using the [TaqMan SNP genotyping assay] (Cat. no. 4351379, Applied Biosystems, ABI, Foster City, CA, USA).

The TaqMan SNP Genotyping Assay contains sequence-specific forward and reverse primers to amplify the polymorphic sequence of interest and two TaqMan minor groove binder (MGB) probes with nonfluorescent quenchers (VIC-labeled probe to detect allele 1 sequence and FAM-labeled probe to detect allele 2 sequence). The context sequence [VIC/FAM]: TGCCCTTCCCATTTTCCTTCAGAAG[A/G] AGAGATTCTTCTATGACCTCATTGG.

According to manufacturers’ instructions, briefly, 1 ul of SNP assay was added to 10 ul of TaqMan Genotyping Master Mix (Applied Biosystems, ABI, Foster City, CA, USA, Cat. no: 4371353), genomic DNA, and the total reaction volume was completed to 20 ul. The PCR procedure included a pre-heating stage of the sample at 60°C for 30 s and 10 min at 95 °C, followed by 40 cycles of thermal cycling. Each cycle consisted of a denaturation step at 95°C for 15 s, followed by annealing and extension at 60°C for 1 min. The process ended with a post-read step at 60°C for 30 s.

### Statistical analysis

Data were computerized and analyzed using the Statistical Package for Social Science (SPSS Inc., Chicago, IL, USA, version 16). Mean and standard deviation (SD) were used to describe quantitative data, while qualitative data were expressed in numbers and percentages. Quantitative data were compared using an independent *t* test when data were normally distributed or Mann–Whitney *U* test when non-parametric data were compared. Kruskal–Wallis test was used when non-parametric data were compared between more than two groups. The chi-square test was used for comparison between qualitative data and Fisher’s exact test was used instead when more than 20% of cells have expected frequencies < 5. The odds ratio (OR) and its 95% confidence interval (95% CI) were calculated to measure the association between variables. Distributions of the genotype were compared with those expected for samples from populations in Hardy–Weinberg equilibrium using the *χ*^2^ test. A *P* value of less than 0.05 was considered significant, and the results were displayed as tables and graphs.

## Results

The study included 80 patients with DR-pulmonary TB in addition to 80 patients with DS-pulmonary TB as a control group.

Table 1 summarized the patient characteristics and clinical history of the two studied groups as there was a significant difference as regard smoking, alcohol intake, drug addiction, and history of contact with TB cases between the two studied groups, while there was no significant difference as regards age, sex, or body mass index and clinical history between both groups.

**Table 1:** Characteristics and clinical history of the studied groups

**Patient characteristic**	**Patients with DR-TB (80)**	**Patients with DS-TB (80)**	**Odds ratio (confidence interval)**	***P* value**
**Age** (mean ± SD)	34.8 ± 5.94	35.9 ± 5.74	-	0.414
**Sex**				
Female	16 (20.0%)	14 (17.5%)	1.18 (0.34–4.17)	0.685
Male	64 (80.0%)	66 (82.5%)		
**BMI (mean** ± SD)	19.82 ± 2.44	19.15 ± 4.55	-	0.480
**Smoking**				
Yes	70 (87.5%)	54 (67.5%)	3.37 (0.96–12.50)	0.002^*^
No	10 (12.5%)	26 (32.5%)		
**Alcohol**				
Yes	24 (30.0%)	8 (10.0%)	3.86 (1.0–16.0)	0.001^*^
No	56 (70.0%)	72 (90.0%)		
**Drug addiction**				
Yes	56 (70.0%)	28 (35.0%)	4.33 (1.5–12.4)	0.000^*^
No	24 (30.0%)	52 (65.0%)		
**Contact of TB case**				
Yes	48 (60.0%)	20 (25.5%)	4.5 (1.57–13.19)	0.000^*^
No	32 (40.0%)	60 (75.5%)		
**Clinical history**				
**Cough**				
Yes	60 (75%.0)	66 (82.5%)	-----	0.246
No	20 (25.0%)	14 (17.5%)		
**Hemoptysis**				
Yes	30 (37.5%)	38 (47.5%)	------	0.200
No	50 (62.5%)	42 (52.5%)		
**Toxic symptoms**				
Yes	62 (77.5%)	54 (67.5%)	--------	0.156
No	18 (22.5%)	26 (32.5%)		

*DR-TB* Drug-resistant tuberculosis, *DS-TB* Drug-sensitive tuberculosis, *BMI* Body mass index

^*^Significant difference (*p* value < 0.05)

The drug resistance pattern in patients of the DR-TB group showed single rifampicin resistance in 40 (50%) of patients and MDR-TB in 40 patients (50%), based on the subsequent results of anti-tuberculous drug susceptibility testing.

There was no significant difference between the two studied groups regarding kidney functions, liver enzymes, complete blood count , and ESR, but there was a significant difference between them regarding serum IL-17 concentration, as demonstrated in Table 2.

**Table 2:** Laboratory and X-ray findings in the studied tuberculous patients

**Laboratory findings** (mean ± SD)	**Patients with DR-TB (80)**	**Patients with DS-TB (80)**	***P* value**
**Urea** (mg/dl)	35.20 ± 15.17	28.93 ± 8.85	0.056
**Creatinine** (mg/dl)	0.80 ± 0.23	0.81 ± 0.22	0.865
**AST** (u/l)	38.90 ± 29.04	31.50 ± 15.51	0.223
**ALT** (u/l)	43.56 ± 36.00	31.00 ± 17.62	0.091
**RBCs** (× 10 ^6^/l)	4.36 ± 0.70	4.33 ± 0.64	0.863
**HB** (gm/l)	10.72 ± 0.98	11.21 ± 1.20	0.091
**Lymphocyte** (× 10 ^3^/ul	35.41 ± 13.86	30.81 ± 8.24	0.124
**TLC** (× 10 ^3^/ul)	6.97 ± 2.87	7.21 ± 2.27	0.722
**Platelets** (× 10 ^3^/ul)	226.0 ± 8179.0	228.0 ± 47737.3	0.904
**ESR** (mm/hour)	94.36 ± 27.54	83.36 ± 23.67	0.103
**IL-17** (pg/ml)	12.61 ± 9.39	5.98 ± 3.07	0.000^*^
**X-ray findings**			
**Infiltrate**	13 (32.5%)	12 (30%)	0.809
**Consolidation**	12 (30%)	18 (45%)	0.165
**Cavitation**	19 (47.5%)	2 (5%)	0.000^*^
**Fibrosis**	4 (10%)	8 (20%)	0.210
**Pleural affection**	0 (0.0%)	4 (10)	0.115
**Area of lesion**			
**No lesion**	- 0 (0.0%)	- 12 (15%)	0.000^*^
**Small**	- 12 (15%)	- 24 (30%)	
**Medium**	- 28 (35%)	- 28 (35%)	
**large**	- 40 (50%)	- 16 (20%)	

*Abbreviations: DR-TB* Drug-resistant tuberculosis, *DS-TB* Drug-sensitive tuberculosis, *AST* Aspartate transaminase, *ALT* Alanine transaminase, *RBCs* Red blood cells, *HB* Hemoglobin, *TLC* Total leucocytic count, *ESR* Erythrocyte sedimentation rate, *IL-17* Interleukin 17

^*^Significant difference (*p* value < 0.05)

The X-ray findings showed more significant cavity formation and large lesion size in the DR-TB group, while the other findings were insignificant between both groups.

Regarding the genotype analysis of IL-17 − 197 G > A (rs2275913) SNP, there was a statistically significant association of GA and AA genotypes with DR-TB patients (*P* < 0.05) as shown in Table 3.

**Table 3:** The distribution of IL-17 − 197 G > A (rs2275913) SNP genotypes and alleles between the studied groups

IL 17 − 197 G > A (rs2275913) SNP	**Patients with DR-TB (80)**	**Patients with DS-TB (80)**	**OR (95% CI)**	***P* value**
**Genotype**				
GG (wild genotype)	38 (47.5%)	60 (75.0%)	0.00 (reference)	
GA	32 (40.0%)	18 (22.5%)	2.81 (0.93–8.64)	0.003^*^
AA	10 (12.5%)	2 (2.5%)	7.89 (0.78–193.14)	0.003^*^
GA + AA (dominant inheritance model)	42 (52.5%)	20 (25.0%)	3.32 (1.17–9.60)	0.000^*^
	*P* = 0.000^*^			
**Allele**				
A	52 (32.5%)	22 (13.8%)	3.02 (1.29–7.19)	0.000*
G (wild genotype)	108 (67.5%)	138 (86.2%)		

*DR-TB* Drug-resistant tuberculosis, *DS-TB* Drug susceptible tuberculosis, *OR* Odd’s ratio, *CI* Confidence interval

^*^Significant difference (*p* value < 0.05)

There was a significant difference between the allelic distribution among the two studied groups (*p* value < 0.05), with a higher frequency of the A allele among the DR-TB group as shown in Table 3.

**Fig. 1:**
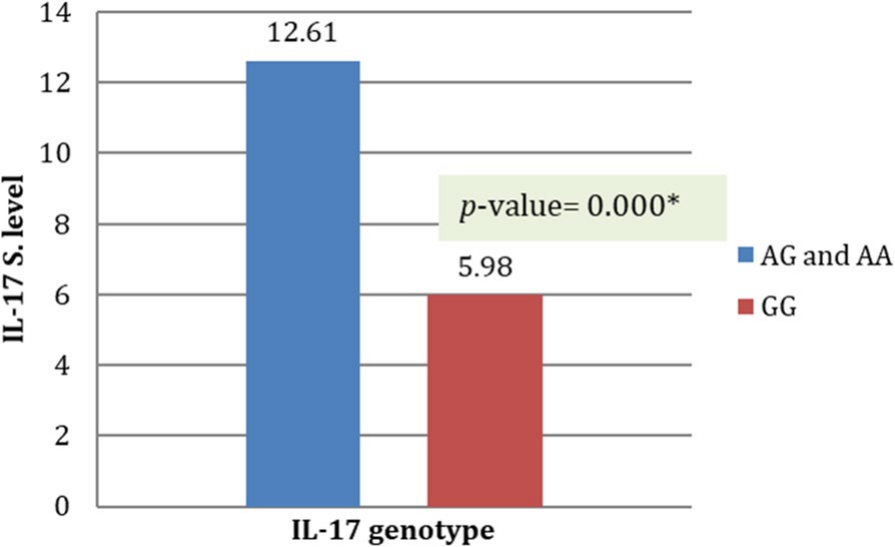
The mean IL-17 level among different genotypes

The mean circulating level of IL-17 was significantly higher in patients with AA and GA genotypes (in both dominant AA + GA (12.61 ± 9.93) and recessive (15.87 ± 10.42) models) than the GG genotype (5.98 ± 3.07) of IL-17 − 197 G > A (rs2275913) SNP as shown in Table 4 and Fig. 1.

**Table 4:** The mean IL-17 level among different genotypes

**SNP/IL-17**	**GG**	**AG**	**AA**	**Kruskal–Wallis Test**	***p* value**
**IL-17 level (pg/ml)** Mean ± SD	5.89 ± 3.07	11.83 ± 9.87	15.87 ± 10.42	Chi-square = 11.773	0.003^*^

^*^Significance difference (*p* value < 0.05)

## Discussion

This study involved 80 patients with DR-pulmonary TB, and 80 patients with DS-pulmonary TB as a comparative group. No significant difference was found between the two groups in terms of the gender of participants (*p* = *0.685*). However, the male gender was higher in both groups representeing (80%) of DR-TB patients and 66% of DS-TB patients, which aligns with the results reported in several other studies [3, 20, 21]. This matches the epidemiological pattern of tuberculosis as males have more hours of outdoor exposure and usually have more risk factors, such as smoking and alcohol intake.

In our study, there was also an insignificant difference between both groups regarding age (*p* = *0.414*). However, the mean age for TB in both groups was similarly reported in other studies [20–22]. These findings were attributed to the high mobility and social interaction during this reproductive age [23].

This study showed a significant difference between DR-TB and DS-TB in terms of smoking, alcohol intake, and drug addiction, which was also reported in previous studies [22, 24, 25].

According to the results of TB drug resistance types, our study revealed that 50% of DR-TB patients were MDR-TB. Higher frequencies were reported by El Hamdouni et al. (73.3%) and Meressa et al. (76%) [21, 26]. This difference may be attributed to the eligibility of exclusion criteria, such as HIV infection and previous TB treatment which compromise essential risk factors for DR-TB.

The pathogen interaction with the host immune system is a key factor promoting the susceptibility, introduction, and progression in patients with TB [8]. Earlier studies on IL-17 focused on its role in inflammation, allograft rejection, autoimmune disorders, and cancer susceptibility, owing to its proinflammatory property [27–29]. Moreover, IL-17 was reported to have a role in bacterial and fungal infections [30, 31].

In MTB, it was demonstrated that IL-17 has a vital role in the initial immune responses and participates in both immune protection and pathology [32–34].

In the current study, we assessed the serum level of IL-17 in patients with DR-pulmonary TB compared to its level in patients with DS-pulmonary TB. Our study showed that the IL-17 levels were significantly higher in the DR-TB group compared to the DS-TB group (*p* < 0.001). These findings are in line with a previous study by Basile et al., who found that T cells from patients with MDR-TB produced higher levels of IL-17 upon stimulation with the MDR strain M (Haarlem family) of MTB than drug-susceptible strains of the same families. They attributed these findings to variations in cell-wall structure between the different strains of MTB, leading to their different binding to pattern recognition receptors on antigen-presenting cells [35].

These higher levels of IL-17 in the DR-TB group may explain the significant presence of cavitation and the larger lesion sizes demonstrated by the X-ray in our study. The IL-17 induces the activation and recruitment of neutrophils, macrophages, and Th1 lymphocytes to the infection site trying to limit the infection and inhibit MTB growth; however, it was reported that IL-17 stimulates apoptosis of alveolar epithelial cells and LDH production with subsequent enhanced pathological damage [15]. Furthermore, IL-17 drives the production of matrix metalloproteinase 3 (MMP-3) from the airway stromal cell aiding in tissue destruction in TB [36].

These results support the idea that IL-17–producing T cells play an immunopathological role in DR-TB, causing significant damage to tissues and possibly contributing to the poor efficacy of second-line drugs on the TB strain [9]. Although the active lung lesions as consolidation and infiltration and the pleural affection were reported to be associated with DR-TB in other studies [23, 37], they were not evident among DR-TB group in our study. This may be explained by the short disease duration and the enrollment of recently diagnosed patients in our study.

In contrast to our results, increased Th17 lymphocytes in the peripheral blood was detected in patients with both drug-susceptible and DR-TB regardless of its clinical form and was associated with high in-vitro IL-17A production [38].

Gene polymorphisms in cytokine genes influence the production of certain cytokines. The rs2275913 SNP, at –197 position from the start codon of the IL17A gene may influence its transcription due to its effect on the gene promoter [39].

Several studies have found a correlation between TB strains susceptibility in general and various cytokine and interleukin gene polymorphisms [40–44]. However, few researchers studied the connection between these polymorphisms and susceptibility to DR-TB strains.

In the current study, there was a significant difference in the frequencies of IL-17 − 197 G > A (rs2275913) SNP genotypes and alleles between the two studied groups. The frequencies of GA/AA genotypes in the DR-TB group (52.5%) were more statistically encountered than in the DS-TB strain group (25%) (*p* < 0.001). Therefore, the AG and AA genotypes of IL-17 − 197 G > A (rs2275913) SNP conferred a significant risk of developing DR-TB (OR = 3.32). The frequency of the A allele was significantly (*p* < 0.001) higher in the resistant group (32.5%) than in the susceptible group (13.8%) which suggests that individuals with the A alleles at IL-17 − 197 G > A (rs2275913) SNP are at higher risk to get DR-TB three times than individuals having the G allele (OR 3.02).

Butov and his colleagues studied polymorphisms of IL-2, IL-4, and IL-10 in MDR-TB and reported that variations in the gene promoter regions of these genes led to changes in the transcription of these interleukins with subsequent effects on their circulating levels [8].

In the current study, there was a significant association between the AG/AA genotypes and the circulating level of IL-17 (*p* < 0.001).

Espinoza et al. reported that the in vitro stimulated peripheral blood mononuclear cells obtained from healthy individuals having the A allele of IL-17 − 197 G > A (rs2275913) SNP transcribed higher levels of mRNA and showed a significantly higher production of the IL-17 protein more than those obtained from healthy individuals without the A allele [45].

These findings coincided with the results of other studies [16, 46]. However, Xie et al. revealed that the mutant A-allele carriers of rs2275913 produced lower levels of IL-17 [47]. Furthermore, Bai et al. reported that IL-17 − 197 G > A (rs2275913) SNP seems not to influence the circulating level of IL-17A [48]. These discrepancies may be attributed to differences in patient demographics, genetic background, and sample size of the study participants.

Our study has some potential limitations that should be considered. One of the main limitations is that, due to financial constraints, we only tested one SNP, and the genotyping of multiple SNPs in the IL-17 gene in Egyptian TB patients should be further explored in future studies.

## Conclusion

Our findings demonstrate that the A allele and the AA/GA genotypes of the IL-17 − 197 G > A (rs2275913) SNP are associated with higher serum levels of IL-17 and might be associated with a higher risk of contracting DR-TB strains in comparison to the susceptibility to the DS strains in Egyptian patients with pulmonary TB, raising the concept of screening individuals at a high risk of catching the DR-TB strains for this polymorphism and that medical interventions aimed at regulating circulating IL-17 levels could be used as preventive and therapeutic measures for these patients.

## Data Availability

The datasets used and analyzed during the current study are available from the corresponding author on reasonable request.

## Abbreviations

DR-TB: The drug-resistant TB
SNP: Single-nucleotide polymorphism
DS-TB: The drug-sensitive TB
TB: Tuberculosis
MDR-TB: Multidrug-resistant tuberculosis
INH: Isoniazid
RIF: Rifampicin
XDR-TB: Extensively drug-resistant tuberculosis
WHO: World Health Organization
MTB: Mycobacterium tuberculosis
ELISA: Enzyme-linked immunosorbent assay
SD: Standard deviation
MMP-3: Matrix metalloproteinase 3
